# Long-term Kentucky bluegrass cultivation enhances soil quality and microbial communities on the Qinghai-Tibet Plateau

**DOI:** 10.3389/fpls.2025.1510676

**Published:** 2025-03-24

**Authors:** Sida Li, Zhenghai Shi, Wen-hui Liu, Wen Li, Guoling Liang, Kaiqiang Liu

**Affiliations:** ^1^ Key Laboratory of Superior Forage Germplasm in the Qinghai‐Tibetan plateau, Qinghai Academy of Animal Science and Veterinary Medicine, Qinghai University, Xining, China; ^2^ Laboratory for Research and Utilization of Qinghai Tibet Plateau Germplasm Resources, Xining, Qinghai, China

**Keywords:** Qinghai-Tibet Plateau, cultivated grassland, soil microbial communities, soil quality, Kentucky bluegrass

## Abstract

**Introduction:**

Nature-based Solutions (NbS) provide a comprehensive strategy for environmental management, focusing on the protection, sustainable use, and restoration of natural and modified ecosystems. Cultivated grasslands are a form of NbS, offering benefits such as increased biodiversity, improved soil fertility, and greater ecosystem resilience. They are widely acknowledged for their positive impact on restoring degraded grasslands. Kentucky bluegrass (Poa pratensis L.) is widely used for restoring degraded grasslands on the Qinghai-Tibet Plateau. However, long-term cultivation of Kentucky bluegrass can lead to above-ground degradation, which challenges its effectiveness in restoring ecosystem health.

**Methods:**

This study investigates the impacts of Kentucky bluegrass cultivation on soil quality, focusing on soil nutrients, enzyme activities, and microbial communities across different recovery stages. Field experiments were conducted to analyze soil quality dynamics during early (2nd year), mid (6th year), and late (10th year) succession stages of cultivated grasslands on the Qinghai-Tibet Plateau. Our results show that in the early and mid-stages, soil total nitrogen, total phosphorus, and organic carbon storage were significantly lower compared to undegraded grasslands, with the lowest soil quality observed in the early stage (P< 0.05). However, by the late stage, soil quality significantly improved, with total nitrogen, total phosphorus, and organic carbon contents exceeding those of undegraded grasslands by 14.59%. These improvements were driven by enhanced microbial community dynamics and increased nitrogen and carbon cycling enzyme activities, which promoted nutrient utilization and organic matter decomposition. This process was accompanied by a rise in microbial diversity, supporting soil resilience and ecosystem function. Soil total nitrogen emerged as a key determinant of soil quality in both natural and cultivated grasslands, and appropriate nitrogen fertilization strategies were found to effectively enhance grassland productivity and ecosystem health.

**Discussion:**

Overall, this study highlights the potential of Kentucky bluegrass in restoring degraded grasslands by improving soil fertility and microbial community structure over time, providing insights into sustainable management practices to maintain soil fertility and ecosystem services on the Qinghai-Tibet Plateau.

## 1 Introduction

The Qinghai-Tibet Plateau serves as the source of major rivers in Asia and is recognized as one of the most biodiverse regions in China and the world. It plays a crucial role in global and regional climate, hydrology, and ecosystem dynamics (Zhang Q, et al., 2022; Liu et al., 2023; Lin et al., 2021). However, in recent years, the grasslands of the Qinghai-Tibet Plateau have experienced varying degrees of degradation, including the emergence of severely degraded grasslands such as “black soil beach” (Zhao et al., 2020; Dong et al., 2015; Zhu et al., 2010). Currently, severe and extreme degradation of grasslands is widespread across the entire plateau. The loss of vegetation cover in these degraded grasslands has resulted in severe soil erosion, the spread of noxious weeds, and conflicts between grassland and livestock (Zhang et al., 2024b). This has had a significant negative impact on the region’s ecology, productivity, and socio-economic conditions (Lin L. et al., 2023; Xu Z. et al., 2023). To address the issue of severe grassland degradation on the Qinghai-Tibet Plateau, China has implemented extensive ecological conservation projects. Among these, Nature-based Solutions (NbS) have gained increasing attention for their holistic and sustainable approach to environmental challenges. NbS focus on protecting, sustainably managing, and restoring natural or modified ecosystems to address societal issues such as climate change, biodiversity loss, and land degradation. These solutions offer multiple benefits, including enhancing ecosystem resilience, improving soil and water quality, and supporting biodiversity (Hua et al., 2022). Currently, it is widely recognized that establishing cultivated grasslands using local plant species is an effective NbS measure. Cultivated grasslands involve the rapid establishment of vegetation cover through the cultivation of adaptive and fast-growing plant species (Cao et al., 2021; Mosier et al., 1991). This approach not only quickly restores vegetation cover but also significantly improves soil conditions. By enhancing soil structural stability and enriching its physical and chemical properties, cultivated grasslands effectively combat soil erosion and promote soil health (Song et al., 2023; Norderhaug et al., 2023; Kavana et al., 2024). Moreover, they provide sustained ecological functions, such as nurturing soil microbial communities, creating habitats for diverse species, and supplying food resources. As a Nature-based Solution, cultivated grasslands exemplify the use of natural processes to restore degraded ecosystems while delivering multiple benefits, including enhanced biodiversity and improved soil fertility (Tomazelli et al., 2024; Johansson et al., 2024). This approach represents a sustainable and holistic strategy for mitigating grassland degradation and fostering ecological resilience on the Qinghai-Tibet Plateau.

Kentucky bluegrass (*Poa pratensis* L.) is a rhizomatous perennial grass species that has a wide distribution in the Qinghai-Tibet Plateau region (Wei et al., 2020; Cao et al., 2019). It exhibits excellent growth adaptability and speed, quickly covering the soil surface, effectively preventing soil erosion, improving soil structure, and promoting soil carbon sequestration and moisture retention abilities (Cao et al., 2019; Wei et al., 2020). Compared to other grass species used for ecological restoration, such as *Elymus* spp. and *Festuca Linn.*, Kentucky bluegrass exhibits superior adaptability and growth speed, making it an ideal choice for rapid soil coverage and erosion control (Wang et al., 2009; Palit et al., 2021). These characteristics are particularly beneficial for stabilizing degraded grasslands in the Qinghai-Tibet Plateau region. Kentucky bluegrass is extensively used in the management of severely degraded grasslands and is considered one of the key grass species (Wang et al., 2022; Wang W. et al., 2024). However, after 4-5 years of establishment, cultivated Kentucky bluegrass grasslands are prone to secondary degradation. This process is characterized by an increase in weed species and a decline in the proportion of desirable forage grasses. Additionally, there is a significant reduction in community biomass and soil nutrient content, which further contributes to the overall degradation of the grassland ecosystem (Zhao et al., 2023; Wu et al., 2023). Therefore, secondary degradation is one of the primary issues associated with the establishment and cultivation of Kentucky bluegrass (*Poa pratensis* L.) in grasslands.

Previous studies have extensively explored and experimented with the issue of secondary degradation in cultivated grasslands. For example, Shang (Shang et al., 2018), based on a comprehensive analysis of the progress in research and restoration efforts on degraded black soil patches over the past decade, suggested that the increase in weed species, the decline in the proportion of desirable forage grasses, and the rise in aboveground biomass within cultivated grasslands may indicate the early stages of directional succession in alpine meadows. However, (Song et al., 2018) argued that the short-term high productivity of artificial grasslands often comes at the expense of soil fertility, which negatively impacts the long-term stability of grassland ecosystems. Similarly, (Dong et al., 2013) held the view that the high productivity of cultivated grasslands in the short term relies heavily on the excessive depletion of soil fertility. Based on the theory of stability maintenance, he proposed that interventions such as fertilization, weeding, and rodent control could help sustain the stability of artificial grasslands. In contrast, Li Xilai, through large-scale plot surveys and quantitative modeling, concluded that minimizing human disturbance is crucial for accelerating the recovery of cultivated grasslands from extreme degradation (Li, 2012). In summary, the precise processes underlying the degradation of cultivated grassland ecosystems remain unclear. The lack of targeted and effective conservation and management strategies is a major factor contributing to exacerbated grassland degradation and the decline in ecosystem services. Therefore, gaining a deeper understanding of the degradation processes in artificial grassland ecosystems is of critical importance for developing scientifically sound and effective conservation and management strategies to maintain the health and stability of grassland ecosystems.

Assessing soil quality facilitates timely monitoring of the current status and dynamic changes in soil. This enables sustainable management of cultivated grasslands (Karlen et al., 2003). Current research indicates that under specific climatic conditions in the Qinghai-Tibet Plateau region, the accumulation rate of organic matter is relatively slow, and soil erosion results in severe nutrient loss. Soil nutrients are one of the primary factors limiting plant growth (Yao et al., 2021).Therefore, evaluating soil quality in cultivated grasslands is beneficial for understanding the soil conditions and trends in different stages of grassland evolution, which can facilitate better management and utilization of soil resources. Additionally, soil microorganisms play a vital role in soil and plant health, including nutrient cycling, carbon sequestration, pollutant degradation, plant disease control, and promoting plant growth (Paul et al., 2024). Exploring the soil microbial community in cultivated grasslands can assess various aspects of the soil environment and reveal soil characteristics and changes in grassland ecosystems (Pedrinho et al., 2024). Consequently, this study focuses on soil nutrient content (organic carbon, total nitrogen, and total phosphorus) and soil enzyme activities related to nutrient cycling as the main indicators for evaluating cultivated grassland soils. By analyzing the microbial community in cultivated grassland soils, the study aims to examine soil changes in different successional stages of cultivated grasslands and explore the soil evolution process, providing a basis for maintaining the health and stability of cultivated grassland ecosystems.

Current research has indicated that during the early establishment phase of cultivated grasslands, insufficient vegetation cover and underdeveloped root systems lead to inadequate protection of the soil surface and a reduction in the input of litter and root exudates. These conditions not only weaken the accumulation of soil organic matter and reduce soil fertility but also increase the risk of soil erosion due to the exposure of the soil surface (Li et al., 2025). Concurrently, management practices such as tillage significantly alter the soil’s physical structure, resulting in increased soil bulk density and decreased porosity, which in turn affect soil aeration and permeability. These changes further restrict the habitat for soil microorganisms, reducing their activity and diversity, while accelerating the mineralization of soil organic matter and exacerbating nutrient loss (Liu et al., 2025; Nonxuba et al., 2025). Based on this, we propose the hypothesis that in the early stages of establishment, cultivated grasslands dominated by Kentucky bluegrass will result in a decrease in soil quality. However, as the evolution process of cultivated grasslands progresses, they will gradually approach the soil quality of undisturbed natural grasslands (i.e., climax communities). The main objectives of this study are: (1) to investigate the impact of different successional stages of cultivated grasslands dominated by Kentucky bluegrass on soil quality; (2) to explore the soil factors influencing the establishment of cultivated grasslands dominated by Kentucky bluegrass; and (3) to examine the effects of cultivated grasslands dominated by Kentucky bluegrass on soil microbial communities.

## 2 Materials and methods

### 2.1 Experimental design

A field experiment was conducted to study the changes in soil characteristics in cultivated grasslands in Qinghai Province, China. The experimental site was located near Xi Hai Town, Haibei Prefecture, Qinghai Province (elevation 3,156 m). The region has a plateau continental climate, with an average annual temperature of 0.9 °C. The average annual precipitation is 369.1 mm, and there is no absolute frost-free period. This location is representative of the Qinghai - Tibet Plateau. The high elevation and characteristic plateau continental climate, with its specific temperature and precipitation patterns, are typical of the broader region. Therefore, the selected site can effectively serve as a substitute for the Qinghai - Tibet Plateau in studying the changes in soil characteristics of cultivated grasslands.

**Figure 1:**
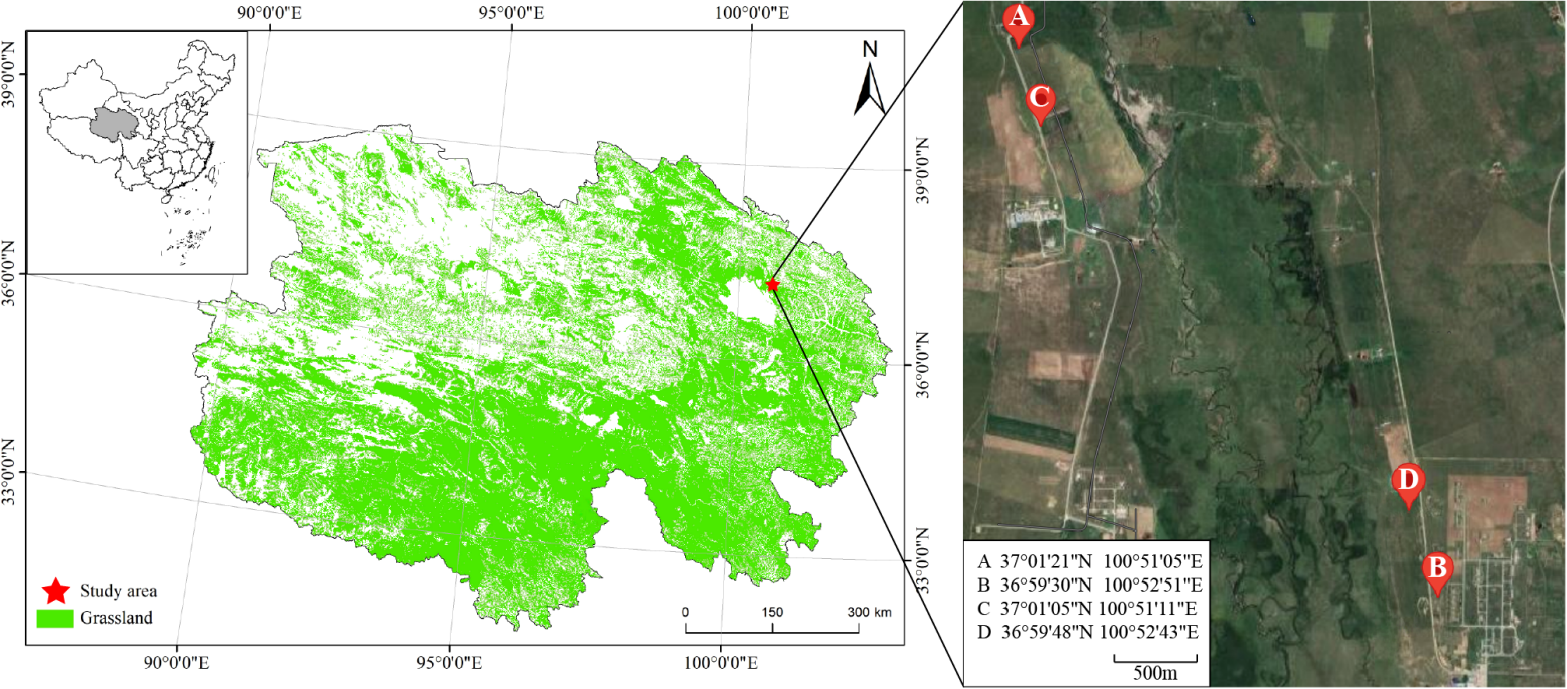
Distribution map of the experimental sites. Point A represents the later stage of restoration (2014). Point B represents the middle stage of restoration (2018). Point C represents the early stage of restoration (2022). Point D represents the un-degraded natural grassland (fenced and protected for 5 years).

Spatial - temporal substitution is a method that uses spatial differences to infer temporal changes. Zhao et al. used this approach to investigate the impact of different cultivation measures on soil quality over time. By comparing grasslands planted at different times but in similar locations, they deduced the temporal changes in soil quality. This method is beneficial because it can effectively eliminate the impact of inter - annual climate variations on soil quality, which are significant in the study region. In this study, a “spatial-temporal substitution” approach was employed. Cultivated grasslands established in 2014, 2018, and 2022 were selected as representative stages of the typical soil succession process (2014 represents the later stage of restoration, 2018 represents the middle stage of restoration, and 2022 represents the early stage of restoration). Additionally, un-degraded natural grassland (fenced and protected for 5 years) was selected as the control (CK) treatment. The slope direction, elevation, and precipitation conditions were similar among the different grassland plots (**Figure 1**).

The cultivation of different grasslands was carried out using strip sowing with a row spacing of 30 cm and a seeding rate of 22.5 kg/hm^2^. Before sowing, urea at a rate of 75 kg/hm^2^ and diammonium phosphate at a rate of 150 kg/hm^2^ were applied, and the cultivation was rainfed. Each plot had an area of 50 m² and was replicated three times. After seeding, one weed removal operation is conducted in the same year, followed by enclosure.

### 2.2 Soil sampling

In 2023, rhizosphere soil samples were collected from cultivated grasslands at different restoration stages of Kentucky bluegrass and from undisturbed natural grasslands. For each treatment, five random replicate samples were collected, and then homogenized to provide a composite sample for each replicate site. Portions of soil were air-dried for characterization analysis, while subsamples for molecular and enzyme activity analysis were preserved with liquid nitrogen, transported in iceboxes, and stored at -80°C and 4°C, respectively.

### 2.3 Soil DNA extraction, PCR amplification, and Illumina sequencing

Using the MagPure Soil DNA LQ Kit (Magan) kit, following the instructions, the genomic DNA of the samples was extracted. The concentration and purity of the DNA were determined using NanoDrop 2000 (Thermo Fisher Scientific, USA) and agarose gel electrophoresis, and the extracted DNA was stored at -20°C. The extracted genomic DNA was used as a template for PCR amplification of bacterial 16S rRNA genes and ITS genes. The V3-V4 variable region of the 16S rRNA gene was amplified using the universal primers 343F (5’-TACGGRAGGCAGCAG-3’) and 798R (5’-AGGGTATCTAATCCT-3’), while the ITS1 variable region of the ITS gene was amplified using the universal primers ITS1F (5’-CTTGGTCATTTAGAGGAAGTAA-3’) and ITS2 (5’-GCTGCGTTCTTCATCGATGC-3’).

The PCR amplification products were analyzed using agarose gel electrophoresis. The AMPure XP beads were used for purification, and the purified products were used as templates for the second-round PCR amplification. Subsequently, another round of purification with magnetic beads was conducted, and the purified second-round products were quantified using Qubit. The concentration was adjusted for sequencing. Sequencing was performed on the Illumina NovaSeq 6000 sequencing platform, generating 250 bp paired-end reads. The sequencing was conducted by Shanghai Oe Biotech Co., Ltd.

After the raw data is sequenced, the first step involves using the Cutadapt software to trim off the primer sequences from the raw data sequences. Subsequently, DADA2 is utilized for quality filtering, denoising, merging, and chimera removal of the paired-end raw data, conducting quality control analysis to obtain representative sequences and abundance tables. Finally, the QIIME 2 software package is employed to select representative sequences of each Amplicon Sequence Variant (ASV) and annotate all representative sequences by comparing them to the Silva database.

### 2.4 Calculation of soil quality index

This study uses Principal Component Analysis (PCA) and correlation analysis to determine the Multidimensional Scaling (MDS) values for soil quality assessment (Zhang Z. et al., 2022). The Norm value is determined by the formula:


$$N_{ik}=\sqrt{\sum_{i}^{k}\left(u_{ik}^{2}\times \lambda_{k}\right)}$$

where Nik is the comprehensive load on the first k principal components with the characteristic value of the ith variable ≥1. Uik is the load of the i-th variable on the k-th principal component, which reflects the importance of the i-th variable in the k principal component. λk is the kth principal component eigenvalue.

The Soil Quality Index (SQI) is calculated through an equation that determines the weights and scores of soil evaluation indicators.


$$W_{i}=P_{i}/\sum_{i=1}^{n}P_{i}\;\left(n=1,\;2,3,4\ldots n\right)$$

$$SQI=\sum_{i=1}^{n}W_{i}\times S_{i}$$

where N is the number of indicators. S_i_ is the membership value of the soil index. P_i_ represents the contribution rate of the i-th comprehensive index for each treatment. W_i_ is the indicator weight (Refer to Tian et al., 2018 for calculation method). The SQI ranges from 0 to 1 (Zhang F. et al., 2022).

Due to the varying impact of different soil physicochemical properties on soil quality, the membership functions for different soil indicators also differ. Membership functions can be classified as ascending and descending types. The types of membership functions for different soil physicochemical indicators are shown in **Table 1**.

**Table 1:** Soil quality iturndicators and membership function types.

Indicator	Membership function type	Membership function formula
Soil bulk density (SBD)	Descending membership function	$\begin{array}{l} f\left(x\right)=\\ \{\begin{array}{c} 1.0\;\;\;\;\;\;\;\;\;\;\;\;\;\;\;\;\;\;\;\;\;\;\;\;\;\;\;\;\;\;\;\;\;\;\;\;\;\;\;\;\;\;\;x=a\\ 0.9\left(b-x\right)/\left(b-a\right)\;\;\;\;a<x<b\\ 0.1\;\;\;\;\;\;\;\;\;\;\;\;\;\;\;\;\;\;\;\;\;\;\;\;\;\;\;\;\;\;\;\;\;\;\;\;\;\;\;\;\;\;\;\;x=b \end{array} \end{array}$
pH		
Catalase (SCAT)	Ascending membership function	$\begin{array}{l} f\left(x\right)=\\ \{\begin{array}{c} 0.1\;\;\;\;\;\;\;\;\;\;\;\;\;\;\;\;\;\;\;\;\;\;\;\;\;\;\;\;\;\;\;\;\;\;\;\;\;\;\;\;\;\;\;x=a\\ 0.9\left(x-a\right)/\left(b-a\right)\;\;\;\;a<x<b\\ 1.0\;\;\;\;\;\;\;\;\;\;\;\;\;\;\;\;\;\;\;\;\;\;\;\;\;\;\;\;\;\;\;\;\;\;\;\;\;\;\;\;\;\;\;\;x=b \end{array} \end{array}$
Cellulase (SCL)		
Alkaline protease (SALPT)		
Alkaline phosphatase (SAKP)		
Urease (SUE)		
Basic xylanase (SBAX)		
Sucrase (SSC)		
Bacteria Shannon		
Fungi Shannon		
Soil organic carbon (SOC)		
Total nitrogen (TN)		
Total phosphorus (TP)		
C Stock		

f(x) is the membership function and x is the measured value of the evaluation index. a, b respectively indicate the minimum and maximum values of the measured values. Since the experimental site of this study is located in the Tibetan Plateau, where the soil pH values are all above 7.5, a higher soil pH is detrimental to plant growth. Therefore, the soil pH is classified as a Descending membership function.

### 2.5 Statistical analysis

The Mothur software package (v.1.30.1) was used to estimate community diversity using the Shannon index and calculate classification α diversity. Non-metric Multidimensional Scaling (NMDS) was employed to describe the clustering of different samples, further reflecting the changes in microbial community structure under intercropping patterns, i.e., microbial diversity. Redundancy Analysis (RDA) was conducted to study the correlations between soil microbial composition, soil nutrients, soil pH, soil bulk density and soil enzyme activities. The RDA analysis was performed using the CANOCO 4.5 software package. Spearman correlation analysis was conducted using IBM SPSS Statistics 19.0 to determine the relationships between soil microbial characteristics (abundance, α diversity, and β diversity) and soil properties (Soil nutrients, soil pH, soil bulk density) and enzyme activities. One-way Analysis of Variance (ANOVA) was used to analyze differences in soil properties (Soil nutrients, soil pH, soil bulk density), soil enzyme activities, and microbial characteristics under different intercropping patterns (P< 0.05). Mean differences were assessed using the LSD test (P< 0.05), where different letters indicate significance. Origin 9.1 was used for data visualization, including soil nutrients and enzyme activities.

## 3 Results

### 3.1 Soil physicochemical properties and soil enzyme activity

**Figure 2:**
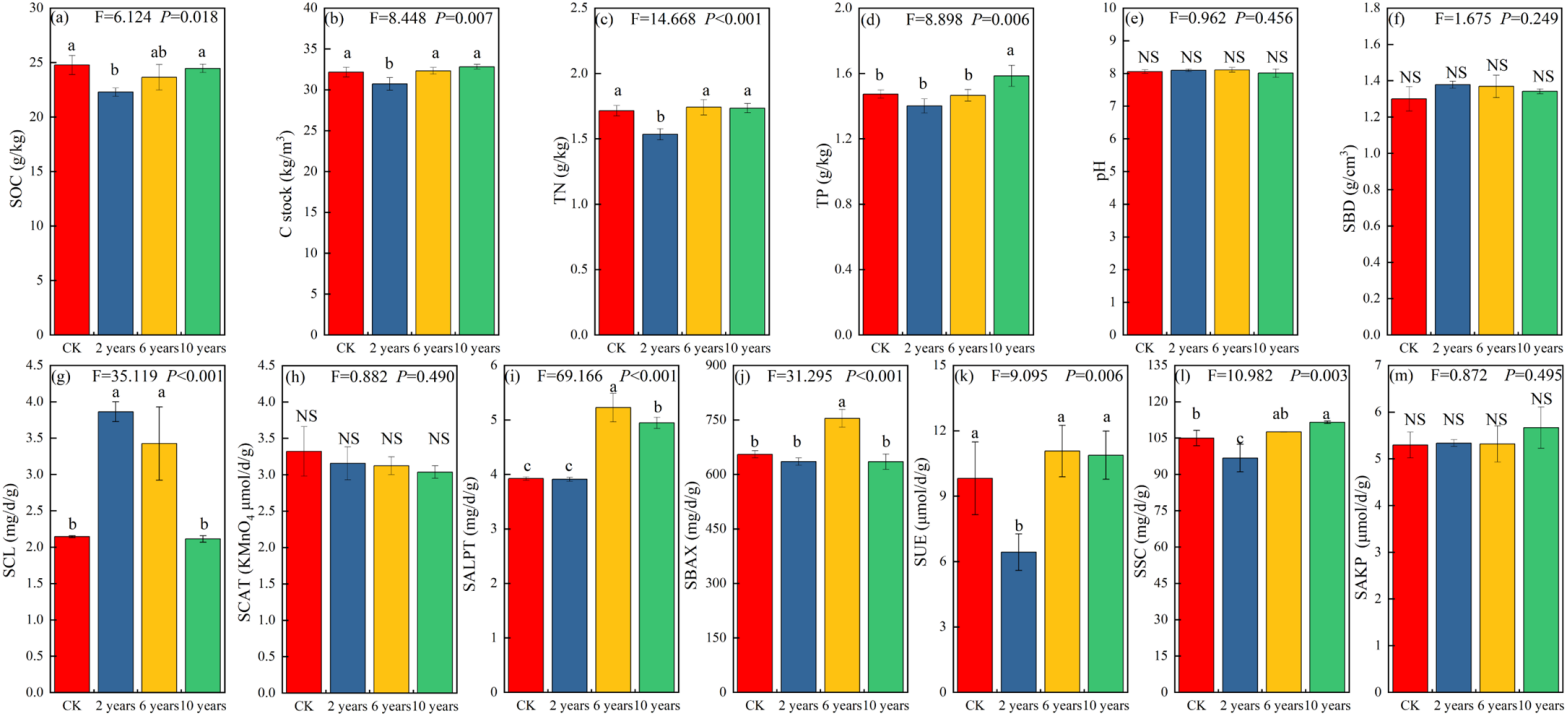
Soil physicochemical properties of different treatments. Different letters indicate significant differences (*P*<0.05). Panel **(a)** represents soil organic carbon content; Panel **(b)** is soil carbon stock; Panel **(c)** is soil total nitrogen content; Panel **(d)** is soil total phosphorus content; Panel **(e)** is soil pH value; Panel **(f)** is also soil pH value; Panel **(g)** is soil cellulase activity; Panel **(h)** is soil catalase activity; Panel **(i)** is soil alkaline protease activity; Panel **(j)** is soil alkaline xylanase activity; Panel **(k)** is soil urease activity; Panel **(l)** is soil sucrase activity; Figure **(m)** is soil alkaline phosphatase activity. Different letters indicate significant differences (*P*<0.05).

The cultivated grassland in different management stages significantly influenced the content of SOC, C stock, TN, and TP (*P*<0.05) (**Figures 2a–f**). In the second year of grassland cultivation (early restoration stage), the content of SOC, C stock, TN, and TP in the cultivated grassland was significantly lower compared to the CK treatment. With the increase in restoration time, the SOC, C stock, TN, and TP of the cultivated grassland showed an increasing trend (**Figures 2c–f**). Gradually, the differences compared to the CK treatment became non-significant (P>0.05) (**Figures 2a–d**).

For soil enzyme activity, the activity of SALPT (alkaline protease), SBAX (asic xylanase), and SUE in cultivated grasslands at different management stages significantly influenced (*P*<0.05), with similar trends of changes in activity. They all showed an increase followed by a decrease with increasing years of management (**Figures 2i–k**). In the second year of grassland cultivation, the activity of SALPT (alkaline protease) did not differ significantly from the CK treatment (*P*>0.05). With the increase in management time, the activity of SALPT (alkaline protease) significantly increased in the sixth and tenth year compared to the CK treatment (*P*<0.05) (**Figure 2i**). As for SBAX, the activity did not differ significantly from the CK treatment in the second and tenth year of management, but the activity in the sixth year of cultivated grassland significantly increased compared to the CK treatment (*P*<0.05) (**Figure 2j**). Regarding SUE, the activity did not differ significantly from the CK treatment in the sixth and tenth year of management, but in the second year of cultivated grassland, the activity of SUE significantly decreased compared to the CK treatment (*P*<0.05) (**Figure 2k**).

In addition, the activity of SCL (cellulase) and SSC (sucrase) in cultivated grasslands with different planting years significantly influenced (*P*<0.05). The activities of SCL (cellulase) and SCAT (catalase) decreased with the increase in management years (**Figures 2g, h**). In the second year of grassland cultivation, the activity of SCL (cellulase) was significantly higher than the CK treatment (*P*<0.05), but with the increase in management time, the activity of SCL (cellulase) did not differ significantly from the CK treatment (*P*>0.05) (**Figures 2g, h**). The activities of SSC (sucrase) and SAKP (alkaline phosphatase) showed an increasing trend with the increase in planting years, but the impact on SAKP (alkaline phosphatase) activity was not significant (*P*>0.05) (**Figures 2l, m**). The activity of SSC (sucrase) decreased the most with the increase in management time, and in the tenth year of cultivated grassland, the activities of SCL (cellulase) and SSC (sucrase) were gradually significantly higher than the CK treatment (*P*<0.05) (**Figure 2l**).

### 3.2 Soil microbial community

**Figure 3:**
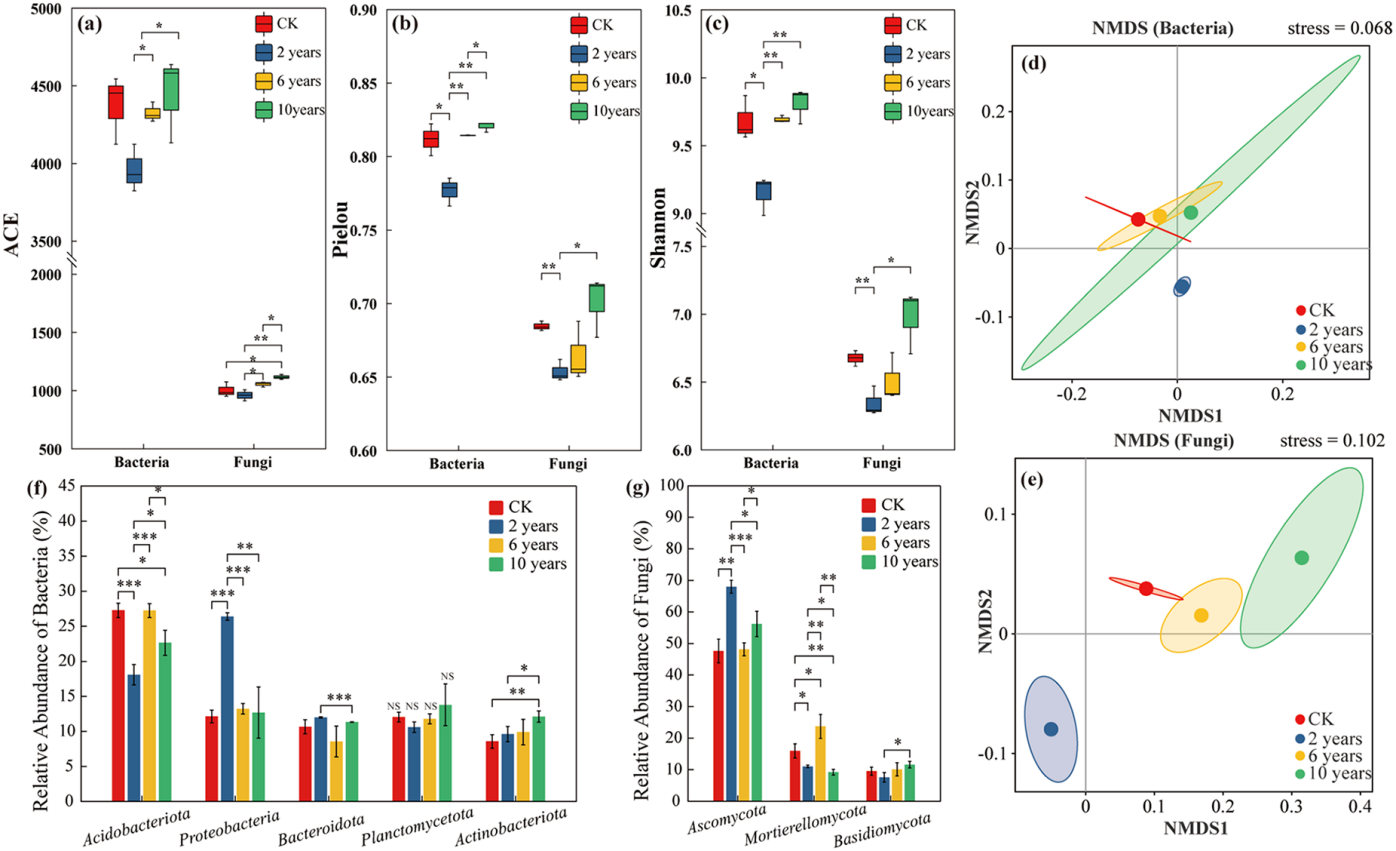
Soil microbial communities of different treatments. “*” in the figure indicates *P* ≤ 0.05, “**” indicates *P* ≤ 0.01, “***” indicates *P* ≤ 0.001. **Panels (a–c)** represent the ACE, Pielou, and Shannon indices of microbial communities under different treatments. **Panels (d, e)** represent the β-diversity of soil bacterial and fungal communities under different treatments. **Panels (f, g)** represent the composition of soil bacterial and fungal communities under different treatments.

In the second year of grassland cultivation, the ACE index of bacterial and fungal communities was significantly lower than the CK treatment (*P*<0.05). However, with prolonged management, the ACE index gradually increased. By the tenth year, the ACE index of the bacterial community no longer showed significant differences compared to the CK treatment (*P* > 0.05), while the fungal community exhibited a significantly higher ACE index than the CK treatment (*P*< 0.05) (**Figure 3a**). Similar trends were observed for the Pielou and Shannon indices. In the second year, both indices were significantly lower than those of the CK treatment (*P*< 0.05). But with increasing management duration, their values gradually increased, and by the tenth year, no significant differences were observed compared to the CK treatment (*P* > 0.05) (**Figures 3b, c**).

Non-metric multidimensional scaling (NMDS) analysis revealed distinct clustering patterns in bacterial and fungal communities over different cultivation durations. The bacterial communities in the CK treatment, sixth-year, and tenth-year cultivated grasslands were relatively similar, while those in the second-year cultivated grasslands differed significantly from the CK treatment and later successional stages (**Figure 3d**). For the fungal community, the CK treatment and sixth-year cultivated grasslands displayed similarity, whereas the fungal communities in the second and tenth years exhibited significant differences compared to both the CK treatment and the sixth-year cultivated grasslands (**Figure 3e**).

Across different management durations, *Acidobacteriota* (23.82%), *Proteobacteria* (16.11%), *Planctomycetota* (12.06%), *Actinobacteriota* (10.06%), and *Bacteroidota* (9.91%) were the dominant bacterial phyla. The relative abundance of *Acidobacteriota* initially increased and then declined with extended management duration, while *Proteobacteria* showed a continuous decline over time. In contrast, *Actinobacteriota* and *Planctomycetota* increased with longer cultivation years. The relative abundance of *Bacteroidota* initially decreased but later increased with cultivation time (**Figure 3f**).

Regarding fungal communities, the dominant phyla were *Ascomycota* (56.93%), *Mortierellomycota* (14.54%), and *Basidiomycota* (9.31%). The relative abundance of *Ascomycota* initially decreased but increased with prolonged cultivation. *Mortierellomycota* exhibited an initial increase followed by a decrease, whereas *Basidiomycota* steadily increased with extended cultivation time (**Figure 3G**).

LEfSe analysis further highlighted the impact of cultivation duration on microbial communities. In the early stages, cultivated grasslands significantly reduced the relative abundance of various bacterial phyla compared to the CK treatment. However, long-term cultivation resulted in minimal differences between cultivated and CK treatments. For fungal communities, the initial establishment of cultivated grasslands had a lesser impact compared to bacterial communities. Over time, multi-year cultivation increased the relative abundance of various fungal phyla compared to the CK treatment (**Supplementary Figures S1**, **S2**).

### 3.3 Soil quality evaluation based on minimum data set

**Figure 4:**
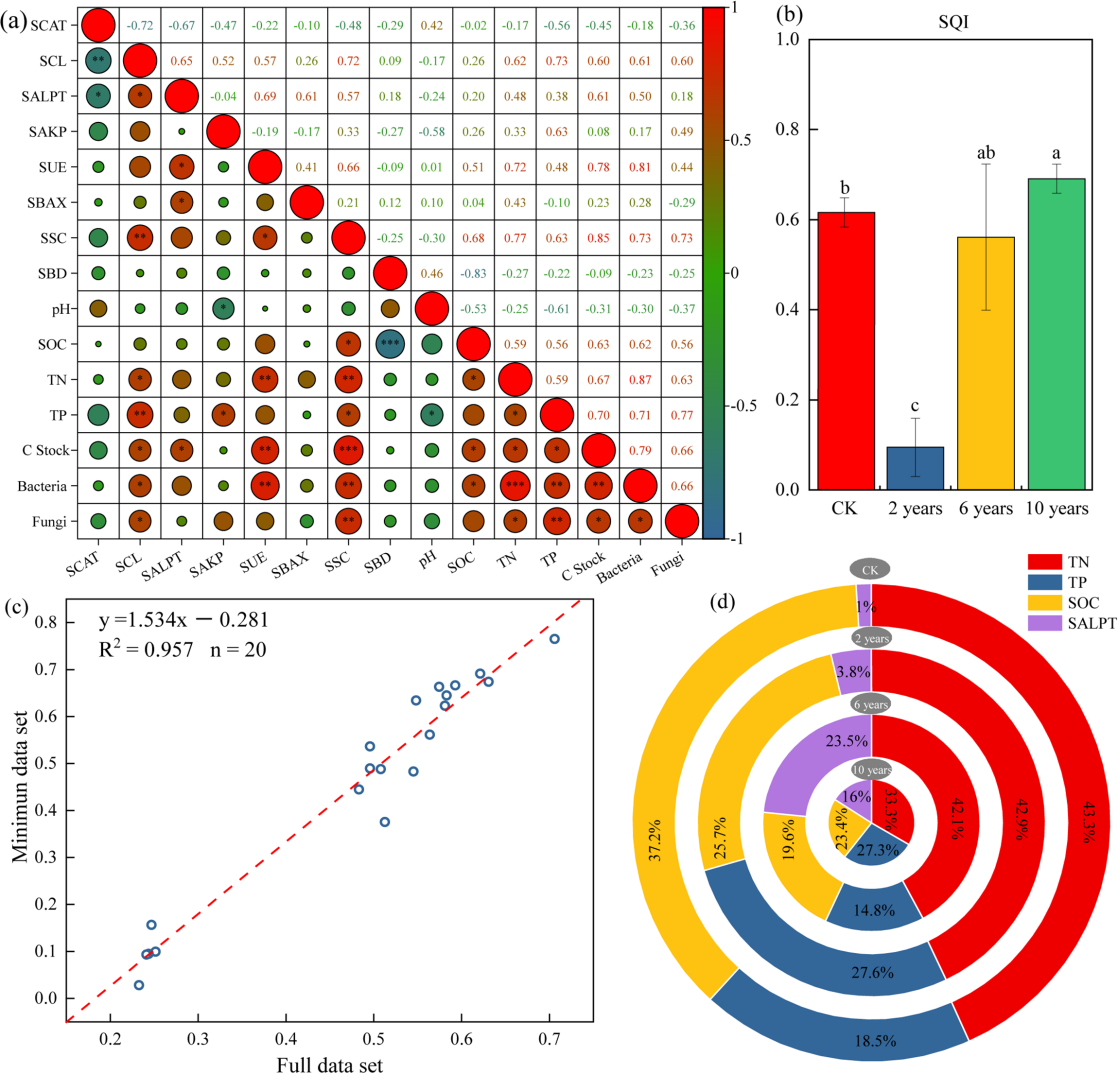
Soil quality index under different treatments. ”*” in the figure indicates *P* ≤ 0.05, “**” indicates *P* ≤ 0.01, “***” indicates *P* ≤ 0.001. **Panel (a)** represents the Pearson correlation of different soil indices. **Panel (b)** represents the soil quality index under different treatments. **Panel (c)** represents the linear relationship of the soil quality index based on MDS and the full data set. **Panel (d)** represents the percentage of different indicators in the soil quality index under different treatments.

Principal Component Analysis (PCA) was performed, and the results are shown in **Table 2**. For each principal component with eigenvalues ≥1, a set of soil indicators was selected based on factor loadings with absolute values >0.5. Indicators with loadings >0.5 in different components were grouped based on low correlation with other indicators (**Figure 4a**). Finally, total nitrogen (TN), total phosphorus (TP), soil organic carbon (SOC), and alkaline protease (SALPT) were selected as the final indicators for soil quality assessment, and PCA was conducted on these indicators (**Table 2**). The calculation of TN, TP, SOC, and SALPT shared common factor variances and weights (**Supplementary Table S1**).

**Table 2:** Soil indicators load matrix and norm value of different treatments.

Indicator	Principal component				Norm	Grouping
	PC-1	PC-2	PC-3	PC-4		
TN	0.908	-0.089	-0.152	0.321	2.298	1
C Stock	0.904	0.216	-0.026	0.162	2.269	1
SUE	0.892	-0.138	-0.013	0.334	2.258	1
Bacteria Shannon	0.878	0.121	-0.197	0.128	2.200	1
SSC	0.784	0.361	-0.127	0.152	2.047	1
SCL	-0.746	-0.126	0.468	0.268	2.017	1
Fungi Shannon	0.729	0.433	-0.084	-0.410	2.028	1
TP	0.655	0.653	-0.114	-0.127	1.972	2
SAKP	-0.035	0.868	-0.186	-0.179	1.525	2
SCAT	-0.247	-0.789	-0.411	-0.257	1.640	2
ph	-0.115	-0.734	0.492	-0.044	1.482	2
SBD	-0.095	-0.080	0.956	0.095	1.483	3
SOC	0.581	0.183	-0.760	0.027	1.864	3
SBAX	0.121	-0.124	0.012	0.927	1.363	4
SALPT	0.531	0.270	0.219	0.710	1.744	4
Eigenvalue	6.059	2.889	2.305	2.009		
Ratio/%	40.396	19.263	15.366	13.393		
Cumulative percentage/%	40.396	59.659	75.025	88.418		

According to the Soil Quality Index (SQI) calculation method, the SQI of different cultivated grasslands and control sites was calculated. The contributions of various evaluation indicators in the Multidimensional Scaling (MDS) analysis were analyzed based on the SQI of different treatments. The study reveals that the SQI of cultivated grasslands shows an increasing trend with the growth of management time, with the SQI of the grassland cultivated in the second year being the lowest. However, as the management time increases, the SQI of the cultivated grasslands gradually increases and in the tenth year (0.69 ± 0.06), significantly higher than the CK treatment (**Figure 4b**). From **Figure 4c**, it can be observed that the soil index of the entire dataset has a good linear relationship with the soil index from the Multidimensional Scaling (MDS) analysis (R^2^ = 0.957, P< 0.01). This indicates that the soil quality assessment indicators based on MDS can serve as a substitute for the entire dataset in evaluating the soil quality of different cultivated grasslands in the study area.

## 4 Discussion

### 4.1 Cultivated grasslands’ impact on soil nutrients and enzyme activity

From the perspective of soil total nitrogen content and nitrogen-cycling related enzyme activities, cultivated grassland succession exhibited distinct patterns across different stages. In the early stages (2nd year), soil total nitrogen content, urease, and protease activities were significantly lower than in undegraded natural grasslands (P< 0.05). This decline is attributed to reduced vegetation cover and aboveground biomass, which limit nitrogen input and accelerate microbial nitrogen decomposition (Xu et al., 2025; Wu et al., 2024; Beniston et al., 2014). These effects are exacerbated by cultivation practices and soil erosion, further reducing soil nitrogen content (Ma Y. et al., 2024, von Lützow et al., 2006; Zhao Y. et al., 2024). However, as succession progresses, soil nitrogen content and enzyme activities (urease and protease) significantly recovered by the 6th and 10th years. This recovery is likely driven by increased nitrogen availability from plant residue decomposition, which enhances soil nitrogen content (De Rosa et al., 2024; Zhu et al., 2023). Additionally, improved soil microbial communities in cultivated grasslands may enhance nitrogen fixation (Xu R. et al., 2023; Ma R. et al., 2024). The increased enzyme activities indicate accelerated nitrogen cycling, benefiting plant nitrogen uptake and soil fertility (Feng et al., 2019; Xue et al., 2018).

Regarding total phosphorus, no significant changes in soil total phosphorus content were observed in the early stages compared to undegraded grasslands (*P*< 0.05). This is likely due to a balance between phosphorus input and consumption during the initial stages of Kentucky bluegrass cultivation (Sun X. et al., 2024). Despite the potential for phosphorus depletion due to cultivation practices and soil erosion, phosphorus levels were maintained through fertilization and soil weathering (Xu et al., 2024). Over time, the decomposition of plant residues continues to release mineral nutrients, increasing soil phosphorus content.

Soil organic carbon (SOC) and carbon stock showed significant reductions in the early stages of grassland succession. This change can be attributed to lower plant diversity, reduced plant input, and higher microbial decomposition rates of organic carbon (Samson et al., 2024). However, in the mid- and late stages of succession, as invasive species increased and plant diversity improved, underground carbon input also increased. This led to the accumulation of soil organic carbon, facilitated by root exudates and microbial activity (Wang A. et al., 2024; Lange et al., 2015). Additionally, changes in sucrase and basic xylanase activities indicate that, over time, the rate of organic matter decomposition in the soil accelerated, promoting soil fertility and microbial activity (Wang A. et al., 2020; Su et al., 2024).

In summary, the succession of cultivated grasslands significantly influences soil nutrients, carbon stock, and enzyme activities. Early succession is characterized by nutrient depletion and low enzyme activity, while longer recovery periods lead to increases in soil nitrogen, phosphorus, and carbon, alongside enhanced enzyme activity.

### 4.2 Impact of cultivated grasslands on soil microbial community

Regarding microbial diversity, soil microbial richness, evenness, and Shannon diversity were lowest in the early stages of cultivated grassland succession. However, with increased recovery time, fungal communities showed significant improvements in richness, evenness, and Shannon diversity, reaching levels comparable to those of undegraded grasslands. In contrast, bacterial communities did not exhibit significant differences compared to natural grasslands. These findings suggest that long-term cultivation of Kentucky bluegrass can restore microbial community diversity and stability, particularly for fungal communities (Sun H. et al., 2024, Li et al., 2024).

Regarding bacterial community changes, in late-stage cultivated grasslands, the relative abundance of specific bacterial taxa shifted significantly. The abundance of *Acidobacteriota* and *Proteobacteria* decreased, while *Actinobacteriota* increased. This shift aligns with the *hypothesis* that increased soil nitrogen levels favor fast-growing bacterial groups such as Actinobacteriota, while slower-growing groups like *Acidobacteriota* and *Proteobacteria* decline (Chen et al., 2024; Pan et al., 2024; Coonan et al., 2020; Zhang et al., 2019). Our results support this trend, with soil nitrogen content increasing over time, accompanied by a decrease in *Acidobacteriota* and *Proteobacteria* and an increase in *Actinobacteriota* (Kielak et al., 2016; Li S. et al., 2024). Additionally, LEfSe analysis revealed that late-stage cultivated grasslands decreased the relative abundance of RB41, *Chthoniobacteraceae*, and *Candidatus Udaeobacter*, while increasing AKYG587. RB41 is involved in nitrogen fixation, and *Chthoniobacteraceae* plays a role in soil polysaccharide decomposition (Zou et al., 2024; Yang et al., 2024). AKYG587 is linked to organic matter decomposition and soil microbial community maintenance (Xing et al., 2020; Arellano-García et al., 2024). These changes suggest that multi-year cultivation of Kentucky bluegrass enhances organic matter decomposition and bacterial community stability (Lin M. et al., 2023; Yusuf et al., 2021).

Regarding fungal diversity, *Mortierellomycota* initially increased but later decreased in abundance. This fungus plays a critical role in mineral phosphorus solubilization and nutrient availability (Liu et al., 2024; Baćmaga et al., 2024). The observed decline may result from a transition from sexual to asexual reproduction, which reduces phosphorus demand over time (Kruczyńska et al., 2023; Wang X. et al., 2024). Consistent with these findings, LEfSe analysis revealed that late-stage cultivated grasslands had lower relative abundances of *Mortierella, Actinomucor, Chaetomiaceae*, and *Fusicolla* compared to natural grasslands. *Mortierella* is involved in soil phosphorus cycling, while *Fusicolla* may form mycorrhizal relationships to enhance nutrient uptake (Ozimek and Hanaka, 2020; Shen et al., 2022).

In summary, the succession of cultivated grasslands promotes the diversity and stability of fungal and bacterial communities, with changes in community composition closely linked to soil nitrogen dynamics and organic matter decomposition.

### 4.3 Impact of cultivated grasslands on soil quality

Current soil quality evaluation methods, such as CASH (Comprehensive Assessment of Soil Health), HSHT (Haney Soil Health Test), and M-SQR (Muencheberg Soil Quality Rating), are widely used for assessing arable soil quality but have limitations when applied to grassland soils (Chang et al., 2022) (.Li et al., 2023; Zhao B. et al., 2024; Yu et al., 2018). Unlike croplands, the grasslands of the Qinghai-Tibet Plateau serve not only to meet livestock production demands but also to achieve ecological value. Therefore, this study assessed the soil quality of cultivated grasslands dominated by Kentucky bluegrass using a range of indicators that reflect ecosystem service functions, including soil bulk density, pH, carbon stock, soil organic carbon (SOC), total nitrogen (TN), total phosphorus (TP), nitrogen and carbon cycling enzymes, and microbial communities.

Results indicate that soil quality in cultivated grasslands improved with increasing recovery time, with late-stage grasslands showing significantly higher soil quality than natural grasslands. This suggests that multi-year cultivation substantially enhances soil quality compared to undegraded natural grasslands (climax communities). Analysis of the Soil Quality Index (SQI) revealed that total nitrogen content was the key factor influencing soil quality, contributing 33.3%–43.3% to the SQI (**Figure 4d**). During the early stages of succession, total phosphorus also contributed significantly to the SQI. These findings highlight the importance of nitrogen and phosphorus fertilization in improving soil quality in both natural and cultivated grasslands. Additionally, the proportion of SOC and TN in the SQI decreased with increasing management time, likely due to their gradual accumulation in the soil, which alleviates their limitations on Kentucky bluegrass growth.

### 4.4 Limitations and future research directions

This study utilized Nature-based Solutions (NbS) to investigate the effects of Kentucky bluegrass-dominated cultivated grasslands on soil quality, nutrient cycling, and microbial community dynamics. Cultivated grasslands, as an NbS, have demonstrated their ability to restore degraded ecosystems while providing multiple benefits, such as enhanced biodiversity and improved soil fertility. However, several limitations of this study highlight the need for further research to optimize the application of NbS in grassland restoration (Bardgett et al., 2021).

Firstly, this study employed a monoculture approach with Kentucky bluegrass. While this method demonstrated substantial improvements in soil quality over longer time scales, monoculture can lead to reduced plant community diversity and compromised stability. Future research should explore mixed sowing strategies that incorporate Kentucky bluegrass with other forage grasses, such as legumes, to enhance plant community diversity and stability while reducing the need for fertilizers.

Secondly, although the study initially explored the impact of Kentucky bluegrass on soil microbial community structure, revealing significant increases in fungal richness and stability, the specific mechanisms by which these microbial changes influence nutrient cycling and ecosystem functions remain unclear. Future research should delve deeper into the functional dynamics of soil microorganisms and the changes in soil metabolites to elucidate their direct and indirect impacts on soil quality.

Lastly, while the study identified soil total nitrogen and phosphorus as key indicators of soil quality, it did not validate the specific effects of fertilization on soil quality. Future research should further investigate the long-term impacts of different fertilization strategies on soil quality in grasslands to optimize management practices and enhance soil health.

In summary, although this study offers preliminary insights into the application of Kentucky bluegrass for grassland restoration, its limitations should not be overlooked. Future research should delve deeper into mixed sowing approaches, soil microbial functions, and the long-term impacts of fertilization strategies. This will help refine the management practices for using Kentucky bluegrass to restore degraded grasslands and enhance overall ecosystem health and sustainability.

## 5 Conclusion

This study demonstrates that Kentucky bluegrass progressively enhances soil fertility and improves microbial community structures over time. Detailed analysis of various stages of grassland recovery revealed that, in the early and mid-stages (2nd to 6th year), soil total nitrogen, total phosphorus, and organic carbon storage were lower compared to undegraded grasslands, with soil quality in the early stage (P< 0.05) being significantly lower. By the tenth year, Kentucky bluegrass substantially increased soil organic carbon, total nitrogen levels, and soil enzyme activities. Compared to pristine natural grasslands, it also significantly supported healthier decomposition capabilities and the resilience of soil microbial communities. As recovery progresses, the soil quality of cultivated grasslands gradually surpasses that of undisturbed natural grasslands. Additionally, soil total nitrogen content emerges as a key determinant of soil quality for both natural and cultivated grasslands on the Qinghai-Tibet Plateau. Implementing appropriate nitrogen fertilization strategies can significantly benefit grassland development and enhance overall ecosystem health.

## Data Availability

The raw data supporting the conclusions of this article will be made available by the authors, without undue reservation.
