# Supplementary material for: Long-term Kentucky bluegrass cultivation enhances soil quality and microbial communities on the Qinghai-Tibet Plateau
Source: Front Plant Sci. 2025 Mar 24;16:1510676. doi: 10.3389/fpls.2025.1510676 (PMC11973338; doi:10.3389/fpls.2025.1510676)
Supplement: Supplementary file 1 [file DataSheet1.docx]

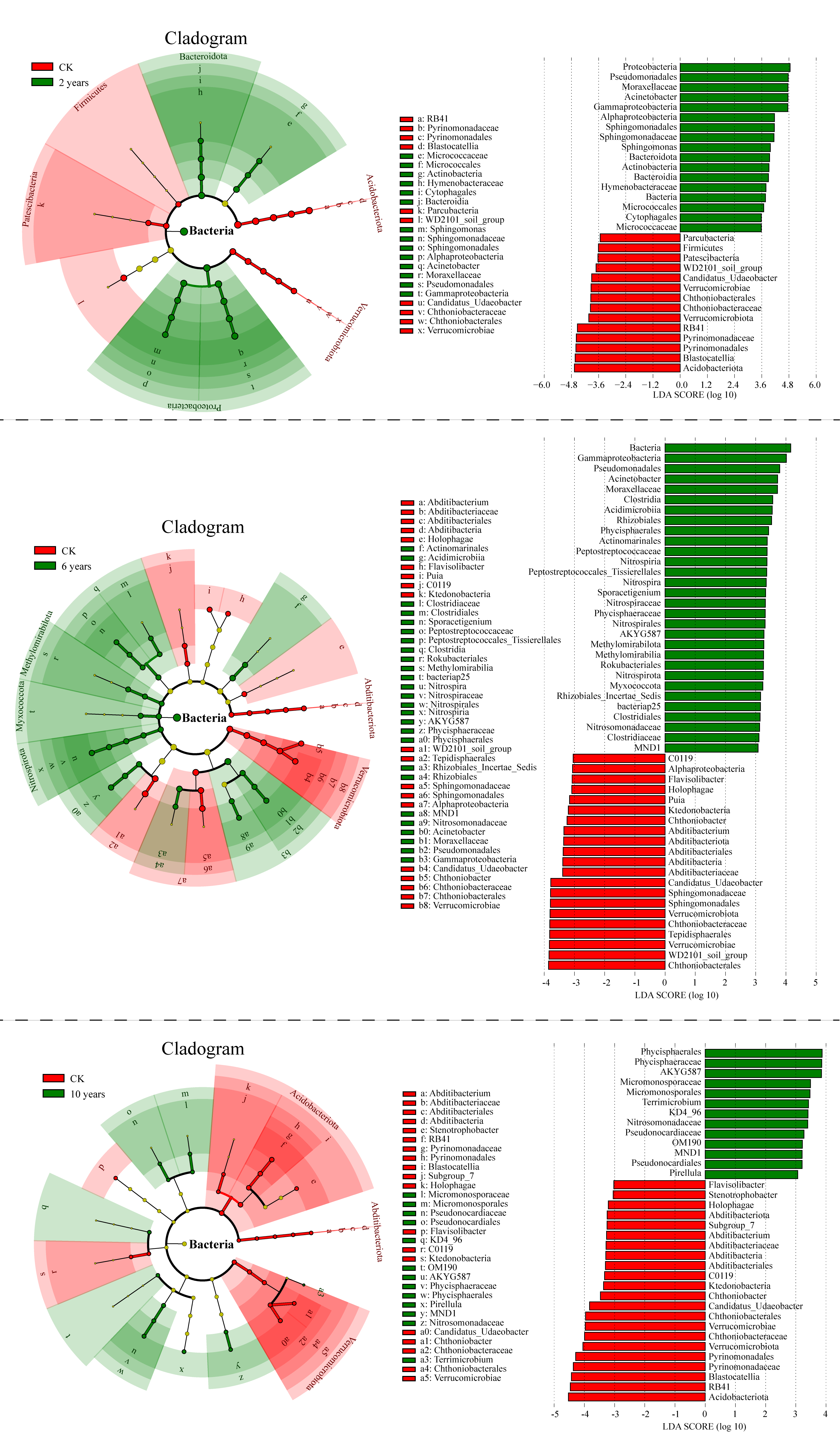


**Fig. S1**

Linear discriminant analysis (LDA) effect size (*LEfSe*) (LDA > 3.5, *P* < 0.05) to identify biomarkers between 0.5% CA treatment and control. The taxa significantly affected by 0.5% CA application are represented by red and green dots, respectively, and the taxa not significantly affected are not shown in the dendrogram. The points from the center to the outer sphere represent the level of phylum, class, order, family and genus.


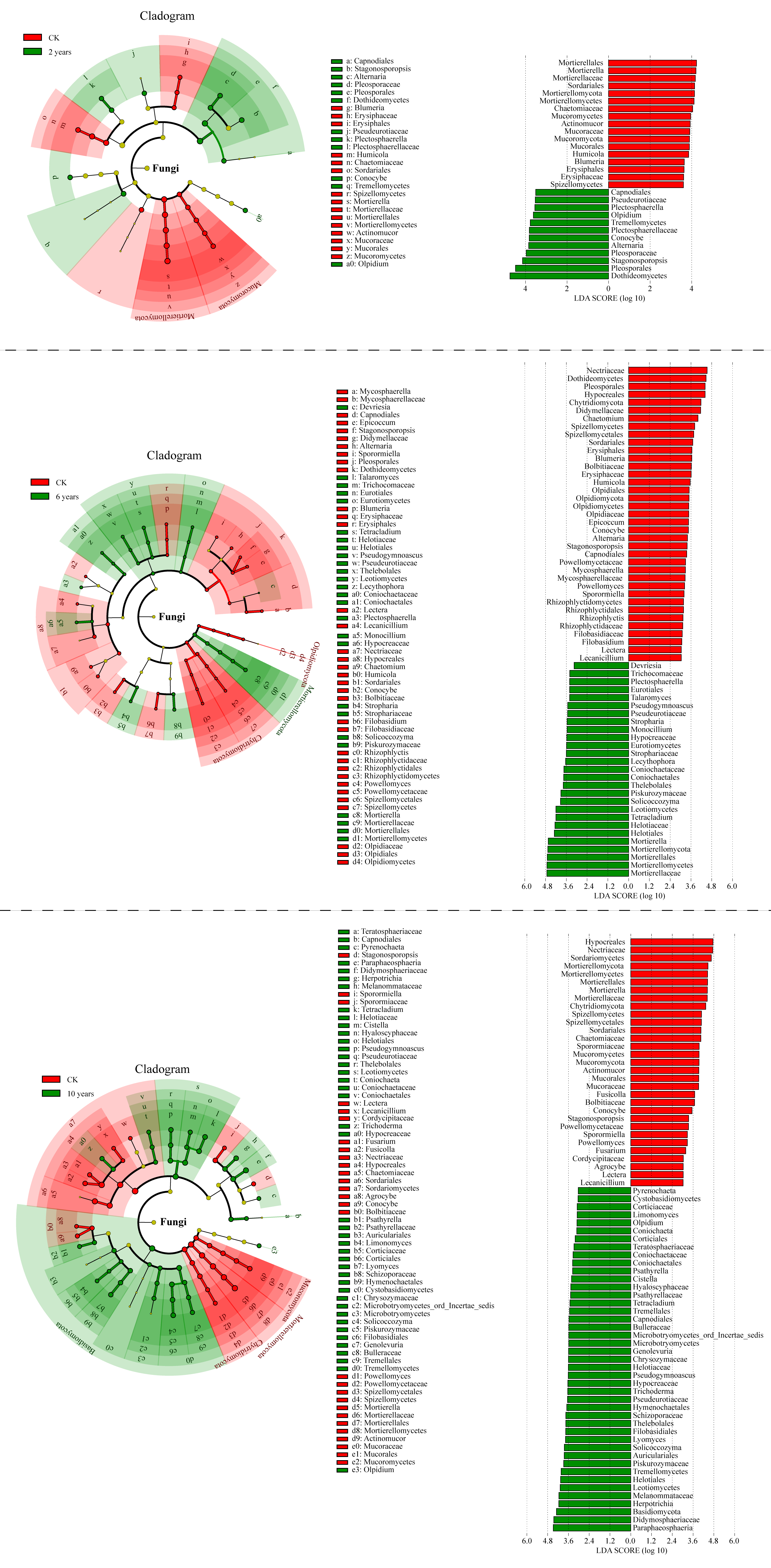


**Fig. S2**

Linear discriminant analysis (LDA) effect size (*LEfSe*) (LDA > 3.5, *P* < 0.05) to identify biomarkers between 0.5% CA treatment and control. The taxa significantly affected by 0.5% CA application are represented by red and green dots, respectively, and the taxa not significantly affected are not shown in the dendrogram. The points from the center to the outer sphere represent the level of phylum, class, order, family and genus.

Table S1 Common factor variance and weight of MDS index.

| Indexed | Common factor variance | Weight |
| --- | --- | --- |
| TN | 0.821 | 28.75% |
| TP | 0.625 | 25.10% |
| SOC | 0.576 | 24.10% |
| SALPT | 0.483 | 22.05% |
